# Prevalence and prognostic significance of malnutrition in diabetic patients with coronary artery disease: a cohort study

**DOI:** 10.1186/s12986-021-00626-4

**Published:** 2021-11-27

**Authors:** Wen Wei, Lingyu Zhang, Guode Li, Zhidong Huang, Jin Liu, Zhihuang Wu, Yuanying Wu, Jinrong Lin, Yunhan Zhang, Yaren Yu, Haozhang Huang, Qiang Li, Bo Wang, Yong Liu, Mei Tu, Hong Chen, Shiqun Chen

**Affiliations:** 1 Department of Cardiology, Guangdong Provincial Key Laboratory of Coronary Heart Disease Prevention, Guangdong Cardiovascular Institute, Guangdong Provincial People’s Hospital, Guangdong Academy of Medical Sciences, Guangzhou, 510080 China; 2 Department of Endocrinology, Longyan First Affiliated Hospital of Fujian Medical University, Longyan, 364000 China; 3 The Second School of Clinical Medicine, Southern Medical University, Guangzhou, 510515 China; 4 Department of Cardiology, Maoming People’s Hospital, Maoming, 525000 China; 5 School of Pharmacy, Guangdong Pharmaceutical University, Guangzhou, 510006 China; 6 Guangdong Medical University, Dongguan, 523808 China; 7 Kunming Medical University, Kunming, 650500 China; 8 Department of Cardiology, The First People’s Hospital of Foshan, Foshan, 528000 China; 9 Guangdong Provincial People’s Hospital, School of Medicine, South China University of Technology, Guangzhou, 510100 China; 10 Department of Endocrinology, Zhujiang Hospital, Southern Medical University, The Second School of Clinical Medicine, Southern Medical University, Guangzhou, 510280 China

**Keywords:** Malnutrition, Diabetic, Coronary artery disease, Prevalence, Prognosis

## Abstract

**Background:**

Malnutrition is associated with poor prognosis in cardiovascular disease patients or in diabetic patients. However, the relationship between malnutrition and clinical outcomes in diabetic patients with coronary artery disease (CAD) is not well known. The aim of this study is to report the prevalence and prognostic consequences of malnutrition in diabetic patients with CAD.

**Methods:**

In this retrospective observational study, the Controlling Nutritional Status (CONUT) score applied to 12,898 consecutive diabetic patients with CAD. The association between malnutrition and long-term all-cause mortality was examined using Cox proportional hazards regression analysis.

**Results:**

According to CONUT score, 60.5% patients suffered from malnutrition; 46.4%, 13.2%, and 0.9% patients had mild, moderate, and severe malnutrition, respectively. During a median follow-up of 4.88 (2.83–7.51) years, 1973 (15.3%) patients died. After adjustment for confounders, malnutrition was associated with significantly increased risk for long-term all-cause mortality (adjusted hazard ratio for mild malnutrition and moderate to severe malnutrition, respectively: 1.38 [95% confidence interval (CI) 1.07–1.77]; P value = 0.012 and 1.63 [95% CI 1.18–2.24]; P value = 0.003). A similar association was observed around subgroups.

**Conclusions:**

Malnutrition is common in diabetic patients with CAD and is strongly associated with increased mortality. It is necessary to adequately assess the nutritional status and take the effective nutritional guidance to improve the prognosis of diabetic patients with CAD.

## Background

Patients with diabetes are at high risk for cardiovascular disease (CVD) [1]. CVD, one of the major macrovascular complications, was a major cause of mortality among diabetic patients, accounting for 50.3% of all deaths. The major contributors was coronary artery disease (CAD), which was responsible for 29.7% [1]. Given the clinical burden that CVD complications have on diabetic patients, there has been an increased focus on the high-risk patients of diabetes with CVD. Identifying high-risk patients based on modifiable clinical characteristics is critical to intervening with these variables to reduce the patient's risk.

Recently, several studies show that malnutrition is correlated with increased in-hospital mortality, long-term mortality and cardiovascular events of acute coronary syndrome (ACS), acute myocardial infarction  (AMI), acute heart failure (HF), chronic heart failure, and atrial fibrillation (AF) [2–8]. Malnutrition is also a significant and common comorbidity in diabetic patients, and it is associated with in-hospital mortality and long-term outcomes [9, 10]. Nutrition is one of the key modifiable risk factors for cardiovascular health in people with or without diabetes [11, 12]. However, the relationship between malnutrition and clinical outcomes in diabetic patients with CAD has not been reported.

Therefore, we aim to assess the prevalence and prognostic consequences of malnutrition in the high-risk patients with both diabetes and CAD using Controlling Nutritional Status (CONUT) score.

## Methods

### Study population

**Fig. 1:**
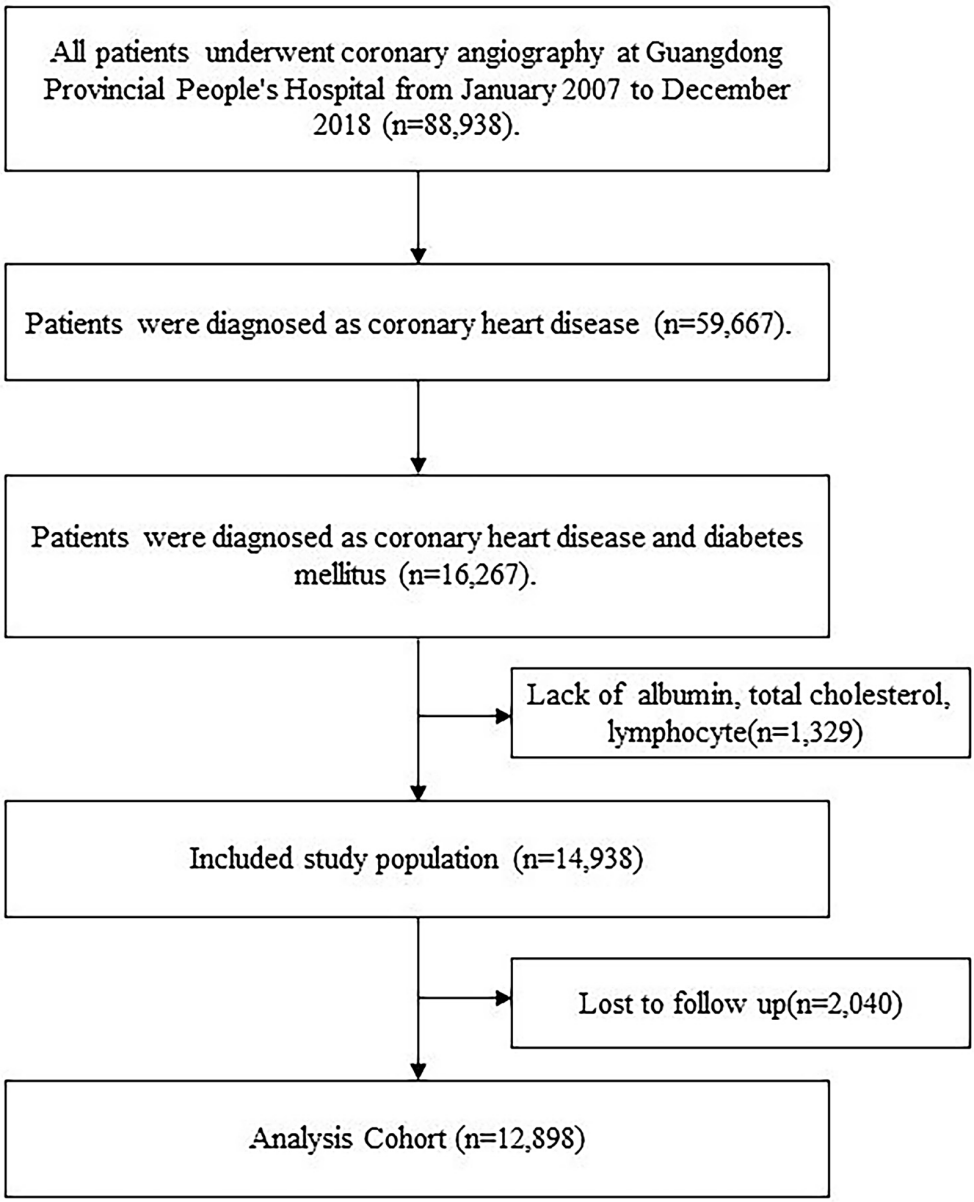
The flow of participants through the trial

The present study was a retrospective observational cohort study, including patients who underwent coronary angiography (CAG) and were diagnosed with both diabetes mellitus (DM) and CAD according to the 10th Revision Codes of the International Classification of Diseases (ICD-10; E10–E14, I20.xx–I25.xx, I50.00001 and I91.40001) at Guangdong Provincial People’s Hospital, Guangdong, China from January 2007 to December 2018 (ClinicalTrials.gov NCT04407936). DM refers to any type of diabetes mellitus, and pregnant women were excluded. CAG or percutaneous coronary intervention (PCI) was performed following standard clinical practice guidelines [13, 14]. Patients without measurement of albumin level, total cholesterol level and lymphocyte count were excluded from this analysis (n = 1329). We also excluded patients with missing data on follow-up (n = 2040). Eventually, 12,898 patients were included (Fig. 1). The study conformed to the principles outlined in the Declaration of Helsinki and was approved by the Guangdong Provincial People’s Hospital ethics committee. All patients gave written informed consent for participation in the study.

### Data collection

Data were extracted from the electronic clinical management records system of the Guangdong Provincial People’s Hospital. The baseline information mainly included demographic characteristics, medical history, medications, laboratory test results and other clinical variables. Data of long-term all-cause deaths were obtained from the Guangdong Provincial Public Security and matched to the electronic Clinical Management System of the Guangdong Provincial People’s Hospital records. Venous blood samples were collected in the early morning after overnight fasting.

### Malnutrition screening tool

We choose the Controlling Nutritional Status (CONUT) score as a screening tool for malnutrition. The CONUT score was developed by Ulibarri et al. [15] in 2005 as a screening tool for the nutritional status of hospitalized patients. It automatically assesses the nutritional status by taking into account serum albumin, total cholesterol and lymphocyte count. A score of 0 to 1 reflects normal; scores of 2 to 4, 5 to 8, and 9 to 12 reflect mild, moderate, and severe malnutrition, respectively.

### Endpoint and follow-up

The primary endpoint was long-term all-cause mortality. Patients were followed up since the date of admission. Follow-up data that were monitored and recorded by trained nurses and research assistants through outpatient interviews and telephones.

### Statistical analysis

Continuous variables were presented as mean (standard deviation [SD]) or medians interquartile range (IQRs), and categorical variables were presented as frequency counts and percentages. One-way analysis of variance (ANOVA) was used to compare the differences in variables among groups. The chi-square test was used to compare proportions between groups.

To assess the association between malnutrition and long-term all-cause mortality, the Cox proportional hazards regression analysis was performed. Variables determining entry into the model were selected based on variables associated with known poor prognosis, clinical plausibility or P value of < 0.05 in the univariate Cox regression analyses. We also performed a subgroup analysis to assess the impact of malnutrition on long-term all-cause mortality. Time-to-event data were presented graphically using Kaplan–Meier (K–M) curves, and a log-rank test was used to assess differences between groups.

All statistical analyses were performed using R, version 4.0.3 software (R Foundation for Statistical Computing, Vienna, Austria). All P values were 2 sided, and values < 0.05 were considered significant.

## Result

### Patient characteristics

A total of 12,898 consecutive diabetic patients with angiographically proven CAD were enrolled. Most patients were men (70.1%), and the mean age was 63.9 ± 10.1 years. Totally, 9748 (75.6%) patients underwent PCI, and more than a quarter had multivessel CAD (26.1%; n = 3367). There were 2361 (18.3%) patients with AMI, 88(3.0%) patients with AF, 1553 (12.0%) patients with congestive heart failure(CHF), 38,825 (68.4%) patients with hypertension, 995 (7.7%) patients with stroke, 3153(24.4%) patients with chronic kidney disease (CKD), and 5066 (39.3%) patients with anemia. Glycosylated hemoglobin (HbA1c) was 7.9% ± 1.7. Fasting blood glucose (FBG) was 9.78 ± 4.62 mmol/L, and 2 h postprandial blood glucose (2hPBG) was 12.83 ± 4.41 mmol/L. Left ventricular ejection fraction (LVEF) was 58% ± 13. More data on the baseline characteristics of study population are shown in Table 2.

### Prevalence and clinical associations of malnutrition

Overall, 7805 (60.5%) patients suffered from malnutrition among the 12,898 patients. By CONUT calculation, 5984 (46.4%), 1703 (13.2%), and 118 (0.9%) patients had mild, moderate, and severe malnutrition, respectively. The prevalence of malnutrition was higher in men than in women (Tables 1, 2).

**Table 1:** Prevalence of malnutrition according to CONUT score

Nutritional Indices	Risk of Malnutrition			
	Absent	Mild	Moderate	Severe
*Formula*				
CONUT, points	0–1	2–4	5–8	9–12
Albumin, g/dl (score)	≥ 3.5 (0)	3.0–3.4 (2)	2.5–2.9 (4)	< 2.5 (6)
Total cholesterol, mmol/l (score)	≥ 180 (0)	140–199 (1)	100–139 (2)	< 100 (3)
Lymphocyte count, *10^9^/l (score)	≥ 1.60 (0)	1.20–1.59 (1)	0.80–1.19 (2)	< 0.80 (3)
Study population, n (%)	5093 (39.5)	5984 (46.4)	1703 (13.2)	118 (0.9)
Male, n (%)	3338 (65.5)	4346 (72.6)	1269 (74.5)	93 (78.8)
Female, n (%)	1755 (34.5)	1638 (27.4)	434 (25.5)	25 (21.2)

*CONUT* Controlling Nutritional Status

**Table 2:** Baseline characteristics of the study population

Characteristic	Risk of Malnutrition					p-value
	Overall	Absent	Mild	Moderate	Severe	
	(n = 12,898)	(n = 5093)	(n = 5984)	(n = 1703)	(n = 118)	
*Demographic characteristics*						
Age (years)	63.9 ± 10.1	62.0 ± 9.9	64.5 ± 10.0	67.1 ± 10.0	67.8 ± 9.9	< 0.001
Female	3852 (29.9)	1755 (34.5)	1638 (27.4)	434 (25.5)	25 (21.2)	< 0.001
*Medical history and Clinical condition*						
T2DM	12,115 (93.9)	4782 (93.9)	5623 ( 94.0)	1598 ( 93.8)	112 ( 94.9)	0.968
T1DM	12 (0.1)	7 (0.1)	4 (0.1)	1 (0.1)	0 (0.0)	0.605
Anemia	5066 (39.3)	1221 (24.0)	2513 (42.0)	1228 (72.1)	104 (88.1)	< 0.001
Stroke	995 (7.7)	322 (6.3)	469 (7.8)	186 (10.9)	18 (15.3)	< 0.001
Hypertension	8825 (68.4)	3429 (67.3)	4106 (68.6)	1201 (70.5)	89 (75.4)	0.028
CKD	3153 (24.4)	789 (15.5)	1476 (24.7)	798 (46.9)	90 (76.3)	< 0.001
CHF	1553 (12.0)	379 (7.4)	686 (11.5)	435 (25.5)	53 (44.9)	< 0.001
AF	388 (3.0)	119 (2.3)	180 (3.0)	83 (4.9)	6 (5.1)	< 0.001
Dialysis history	84 (0.7)	6 (0.1)	23 (0.4)	40 (2.3)	15 (12.7)	< 0.001
AMI	2361 (18.3)	633 (12.4)	1061 (17.7)	616 (36.2)	51 (43.2)	< 0.001
*Coronary vessels involvement*						
Two-vessel disease	128 (1.0)	53 (1.0)	65 (1.1)	8 (0.5)	2 (1.7)	0.112
Three-vessel disease	3365 (26.1)	1230 (24.2)	1572 (26.3)	528 (31.0)	35 (29.7)	< 0.001
Four-vessel disease	2 (0.0)	1 (0.0)	1 (0.0)	0 (0.0)	0 (0.0)	0.952
*Procedure*						
PCI	9748 (75.6)	3793 (74.5)	4517 (75.5)	1344 (78.9)	94 (79.7)	0.002
*Laboratory examination*						
Albumin (g/L)	36.18 ± 4.56	38.93 ± 2.70	35.83 ± 3.74	30.00 ± 3.86	24.54 ± 3.32	< 0.001
Lymphocyte(10^9^/L)	1.94 ± 0.72	2.25 ± 0.63	1.86 ± 0.68	1.36 ± 0.60	0.90 ± 0.38	< 0.001
TG (mmol/L)	1.50 (1.10, 2.14)	1.79 (1.32, 2.58)	1.40 (1.03, 1.91)	1.20 (0.92, 1.63)	1.14 (0.89, 1.47)	< 0.001
TC (mmol/L)	4.45 ± 1.24	5.06 ± 1.08	4.11 ± 1.17	3.89 ± 1.18	3.41 ± 1.05	< 0.001
LDL-C(mmol/L)	2.72 ± 0.96	3.14 ± 0.87	2.47 ± 0.91	2.35 ± 0.92	2.05 ± 0.81	< 0.001
HDL-C(mmol/L)	0.95 ± 0.25	1.01 ± 0.24	0.93 ± 0.24	0.87 ± 0.25	0.75 ± 0.23	< 0.001
CREA(umol/L)	88.10 (73.00, 110.43)	82.53 (69.00, 99.00)	89.10 (74.00,110.00)	108.00 (83.00, 150.00)	150.90 (104.00, 303.30)	< 0.001
eGFR(ml/min/1.73m^2^)	74.90 ± 28.34	81.50 ± 25.84	74.73 ± 27.38	60.31 ± 30.54	41.66 ± 27.63	< 0.001
hs-CRP (mg/L)	4.45 ± 1.24	2.72 (1.04, 6.66)	3.39 (1.04, 9.71)	14.10 (3.65, 40.50)	39.70 (12.92, 78.15)	< 0.001
Hb (g/L)	129.97 ± 18.18	135.77 ± 15.48	129.43 ± 16.99	116.48 ± 19.95	101.54 ± 22.11	< 0.001
HbA1c (%)	7.88 ± 1.70	7.89 ± 1.65	7.86 ± 1.71	7.93 ± 1.80	7.78 ± 1.88	0.572
FBG (mmol/L)	9.78 ± 4.62	9.52 ± 4.31	9.73 ± 4.69	10.57 ± 4.93	12.07 ± 6.61	< 0.001
2hPBG (mmol/L)	12.83 ± 4.41	13.24 ± 4.39	12.67 ± 4.46	12.12 ± 4.12	11.09 ± 5.14	< 0.001
LVEF (%)	57.73 ± 12.74	60.36 ± 11.45	57.37 ± 12.79	51.68 ± 13.76	49.67 ± 13.20	< 0.001
*Treatment during hospitalization*						
OADs	7718 (61.1)	3210 (63.5)	3626 (61.9)	838 (51.9)	44 (42.3)	< 0.001
ACEI/ARB	5807 (46.0)	2363 (46.8)	2688 (45.9)	714 (44.2)	42 (40.4)	0.193
β-blockers	10,400 (82.3)	4247 (84.1)	4803 (82.0)	1266 (78.3)	84 (80.8)	< 0.001
CCB	3398 (26.9)	1333 (26.4)	1569 (26.8)	458 (28.3)	38 (36.5)	0.062
Statins	12,079 (95.6)	4864 (96.3)	5603 (95.6)	1520 (94.1)	92 (88.5)	< 0.001
Aspirin	11,623 (92.0)	4697 (93.0)	5393 (92.0)	1450 (89.7)	83 (79.8)	< 0.001
Diuretic	2203 (17.4)	539 (10.7)	986 (16.8)	620 (38.4)	58 (55.8)	< 0.001

*T2DM* Type 2 diabetes mellitus, *T1DM* type 1 diabetes mellitus, *CKD* chronic kidney disease, *CHF* congestive heart failure, *AF* atrial fibrillation, *AMI* acute myocardial infarction, *PCI* percutaneous coronary intervention, *TG* triglycerides, *TC* total cholesterol, *LDL-C* low density lipoprotein cholesterol, *HDL-C* high density lipoprotein cholesterol, *CREA* creatinine, *eGFR* estimated glomerular filtrationrate, *hs-CRP* hypersensitive C-reactive protein, *Hb* hemoglobin, *HbA1c* glycosylated hemoglobin, *FBG* Fasting blood glucose, *2hPBG* 2 hours postprandial blood glucose, *LVEF* left ventricular ejection fraction, *OADs* oral antidiabetic drugs, *ACEI/ARB* angiotensin-converting enzyme inhibitor/angiotensin receptor blocker, *CCB* calcium channel blocker

According to CONUT score, 12,898 patients were divided into four groups: normal nutritional status, mild malnutrition, moderate malnutrition, and severe malnutrition. Compared with those with normal nutritional status, patients with malnutrition were older, were more likely to be men, and had worse LVEF and renal function. Their FBG level was higher, and hemoglobin (Hb) level was lower than those with normal nutritional status. They also were more likely to have multivessel CAD, AMI, AF, CHF, hypertension, stroke and anemia (Table 2). In addition, the hospital length of stay and hospitalization expenses for the malnourished patients was significantly greater than that for the normal-nourished patients (Table 3).

**Table 3:** Clinical outcomes according to CONUT score

Characteristic	Risk of malnutrition					p-value
	Overall	Absent	Mild	Moderate	Severe	
	(n = 12,898)	(n = 5093)	(n = 5984)	(n = 1703)	(n = 118)	
Cost ($)	8529.6 (5543.2, 12,911.8)	8063.6 (5206.7, 12,173.4)	8617.0 (5560.5, 13,003.4)	9474.6 (6489.0, 14,450.9)	13,415.5 (7535.4, 20,831.4)	< 0.001
Hospital length of stay (days)	5 (3, 8)	4 (3, 7)	5 (3, 8)	7 (4, 11)	12 (7, 21)	< 0.001
In-hospital mortality (%)	99 (0.8)	13 (0.3)	44 (0.7)	35 (2.1)	7 (5.9)	< 0.001
Long-term mortality (%)	1973 (15.3)	533 (10.5)	927 (15.5)	464 (27.2)	49 (41.5)	< 0.001

### Malnutrition and clinical outcomes

**Fig. 2:**
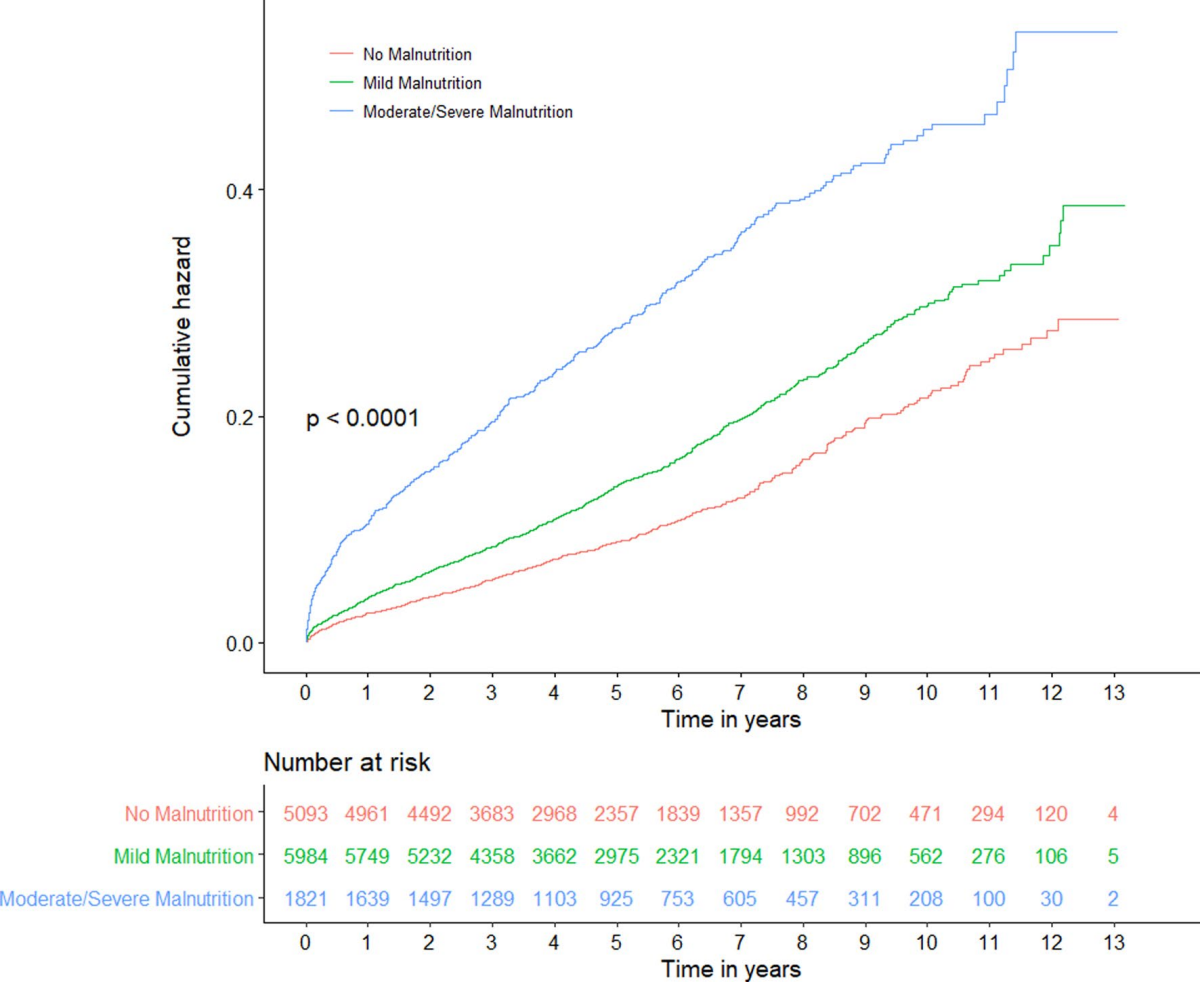
Kaplan–Meier curves for long-term all-cause mortality of malnutrition

During a median follow-up of 4.88 (2.83–7.51) years, a total of 1973 (15.3%) patients died from all causes. During hospitalization, 99 (0.8%) patients died from all causes. The in-hospital all-cause mortality was significantly increased in the malnourished patients (Table 3). Worsening malnutrition status was associated with higher incidence of all-cause mortality (Fig. 2, log-rank test, p < 0.0001).

The Cox proportional hazards regression analysis indicated that compared with normal nutritional status, malnutrition was associated with significantly increased risk for long-term all-cause mortality (adjusted hazard ratio for mild malnutrition and moderate to severe malnutrition, respectively: 1.38 [95% confidence interval (CI) 1.07–1.77]; P value = 0.012 and 1.63 [95% CI 1.18–2.24]; P value = 0.003) (Table 4).

**Table 4:** Cox proportional hazards regression analysis for long-term all-cause mortality

	Univariate		Multivariate	
	HR(95%CI)	p-value	HR(95%CI)	p-value
Mild Malnutrition	1.45 (1.30–1.61)	< 0.001	1.38 (1.07, 1.77)	0.012
Moderate to Severe Malnutrition	2.65 (2.35–2.99)	< 0.001	1.63 (1.18, 2.24)	0.003
Age	1.03 (1.03, 1.04)	< 0.001	1.03 (1.02, 1.04)	< 0.001
Female	0.96 (0.87, 1.06)	0.397	0.79 (0.63, 0.99)	0.04
Hypertension	1.23 (1.11, 1.35)	< 0.001	1.14 (0.90, 1.43)	0.272
Stroke	1.59 (1.38, 1.84)	< 0.001	1.09 (0.79, 1.50)	0.599
CHF	2.85 (2.56, 3.17)	< 0.001	2.17 (1.71, 2.76)	< 0.001
AF	2.05 (1.69, 2.48)	< 0.001	1.63 (1.10, 2.42)	0.015
CKD	2.51 (2.30, 2.75)	< 0.001	1.56 (1.26, 1.93)	< 0.001
Anemia	1.8 (1.65, 1.97)	< 0.001	1.22 (0.99, 1.51)	0.062
Dialysis history	6.07 (4.54, 8.10)	< 0.001	2.33 (1.17, 4.63)	0.016
HbA1c	1.02 (0.99, 1.05)	0.308	1.06 (1.00, 1.12)	0.069
hs-CRP	1.01 (1.01, 1.01)	< 0.001	1.00 (1.00, 1.01)	0.259
HDL-C	0.76 (0.64, 0.91)	0.003	0.98 (0.64, 1.51)	0.935
ACEI/ARB	0.84 (0.77, 0.93)	< 0.001	0.90 (0.74, 1.09)	0.281
β-blockers	0.87 (0.78, 0.98)	0.024	1.31 (1.00, 1.73)	0.055
Aspirin	0.67 (0.58, 0.78)	< 0.001	0.99 (0.72, 1.37)	0.962
OADs	0.83 (0.76, 0.92)	< 0.001	0.98 (0.81, 1.20)	0.877

*CHF* Congestive heart failure, *AF* atrial fibrillation, *CKD* chronic kidney disease, *HbA1c* glycosylated hemoglobin, *hs-CRP* hypersensitive C-reactive protein, *HDL-C* high density lipoprotein cholesterol, *ACEI/ARB* angiotensin-converting enzyme inhibitor/angiotensin receptor blocker, *OADs* oral antidiabetic drugs

**Fig. 3:**
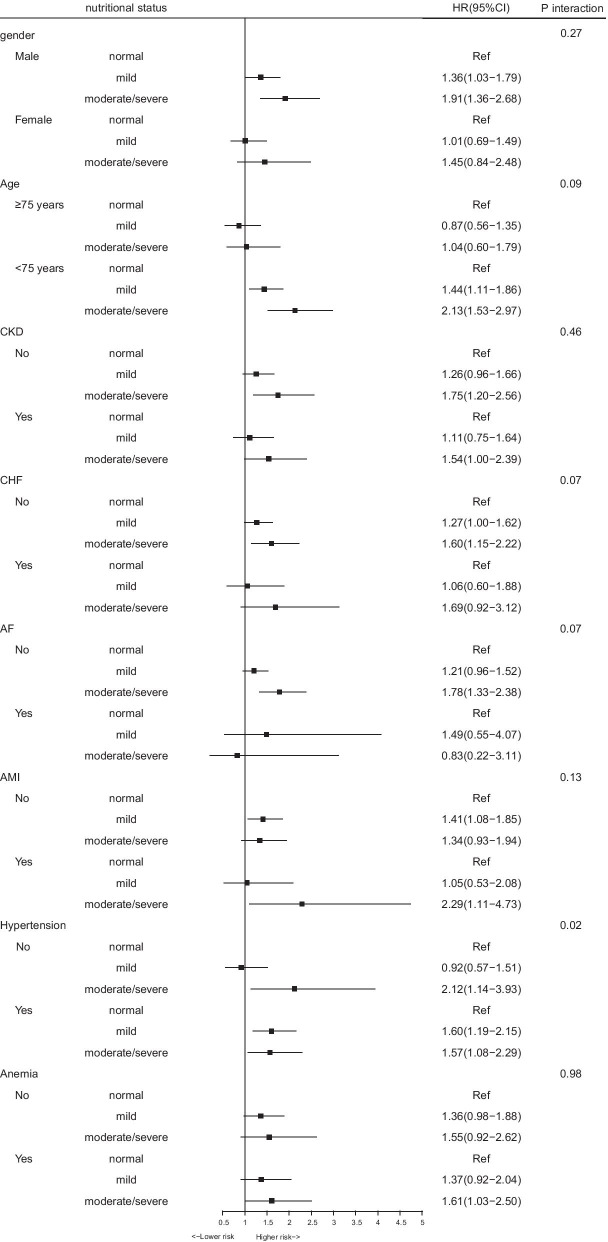
Hazard ratios for long-term all-cause mortality in different subgroups

In a subgroup analysis, the Cox regression analysis revealed that malnutrition had a relatively consistent risk of mortality across dichotomized subgroups (gender, old, CKD, CHF, AF, AMI, and anemia). Significant interaction (P-interaction = 0.02) between malnutrition and hypertension was observed (Fig. 3).

## Discussion

To our knowledge, this is the first study to explore the prevalence and mortality of malnutrition in the high-risk population with both diabetes and angiographically proven CAD. In the study, we found that malnutrition evaluated by CONUT score was common in diabetic patients with CAD. The in-hospital mortality, hospital length of stay, and hospitalization expenses for the malnourished patients was significantly greater than that for the normal-nourished patients. Our research also showed that malnutrition was associated with a poor prognosis regardless of age, HbA1c, AF, CHF, CKD, anemia, and other risk factors. In addition, similar results were found among different subgroups.

Malnutrition was a very common problem in hospitalized elder patients with different diseases [16, 17]. 21% hospitalized elderly patients with diabetes suffered from malnutrition [10]. Roubín SR, et al. reported that the percentage of ACS patients with malnutrition varied from 8.9% with the Prognostic Nutritional Index (PNI), to 49.8% with the CONUT score, and to 59.5% with the Nutritional Risk Index (NRI). The CONUT score showed the highest predictive ability, whereas the NRI had the lowest [3]. Therefore, we chose CONUT score to evaluate nutritional status and found that 60.5% patients with both CAD and diabetes suffered from malnutrition. Hospital malnutrition was associated with an increase in mortality, a higher readmission rate, need of rehabilitation support after discharge and higher healthcare [18–20]. In concordance with the above research, our study indicated that the hospitalization expenses for the malnourished patients was significantly greater than that for the normal-nourished patients. In addition, we reported the in-hospital all-cause mortality and hospital length of stay were significantly increased in the malnourished patients.

There was evidence to indicate worsening of clinical outcomes when diabetes was associated with poor nutritional status, especially in geriatric patients [21]. The risk for mortality in elderly patients with diabetes increased by 69% in malnourished versus normal-nourished patients [9]. Among ACS patients, mild malnutrition increased 36% risk for long-term all-cause mortality, while moderate increased 1.02-fold and severe increased 2.65-fold, respectively than normal nutritional status [3]. Compared with the study in ACS patients, slightly higher risk for mortality was found in the mild malnutrition in our study, but the risk in the moderate to severe malnutrition was lower than in the ACS patients. The reason might be the severity of CAD was lower than the above research due to the proportion of AMI in our study was just 18.3%. Our research showed that the risk for mortality in the moderate to severe malnutrition was slightly lower than the risk in the elderly diabetic patients. This result may be explained in part by different ways of assessing malnutrition and different disease spectrum and age composition of patients. In short, our study found that the more severe the malnutrition, the higher the long-term all-cause mortality, and moderate to severe malnutrition could cause worse prognosis than mild malnutrition. The admission nutritional status was an independent predictor of long-term mortality in diabetic patients with CAD.

Why does malnutrition coexist with DM and CAD in hospitalized patients? This may be related to the effect of the acute disease that led to hospitalization. How does nutritional status influence the prognosis of diabetic patients with CAD remains unclear. One possible explanation is that nutritional status may be a proxy indicator of inflammation [22]. Chronic inflammatory diseases correlate with increased production of catabolic cytokines, muscle catabolism, and appetite suppression and, thereby, lower albumin level [23]. Diabetes is associated with increased systemic inflammation. Furthermore, inflammation is recognized in the pathogenesis of atherosclerotic CVD events [24, 25]. Our study found that the more severe the malnutrition, the higher the hypersensitive C reactive protein level. High degree of malnutrition is associated with high level of inflammation, which translates into increased atherosclerotic burden. The relationship between these 3 entities has recently been described as malnutrition-inflammation-atherosclerosis syndrome [26].

Despite the growing body of studies demonstrating the risk of malnutrition, malnutrition is not commonly listed as a comorbidity of DM and CAD. Our findings strongly support the need for physicians to practice early identification of malnutrition in the high-risk population. Since the variables required for CONUT score calculation are widely available from routine clinical examination, malnutrition might be systematically screened in the DM and CAD setting. Screening these patients might identify patients at high risk of poor outcomes who might benefit from tailored secondary prevention programs with nutritional supplements to improve their prognosis. The interventions should be started during the hospitalization such as nutrition consultation from a dietitian and also continue after discharge to ensure normalization of nutritional status. The present studies demonstrate that mediterranean dietary regimens has a beneficial role in reducing the risk of the incidence and mortality of CVD in population inclusive of individuals with diabetes [12, 27]. Clinicians should keep abreast of current scientific evidence to provide these high-risk patients with effective nutrition guidance. Furthermore, some measures to address chronic inflammation should also be explored.

### Limitation

First, our results were subject to limitations of the observational nature inherent in the retrospectively collected database. Second, we did not compare the prognostic value of nutritional screening tools with more complex comprehensive nutritional screening tools, and we did not investigate the changes in nutritional status over time and their association with outcomes. Third, long-term all-cause mortality was complex and multivariable. Due to the lack of other endpoint events, this will limit to generalize our results. Finally, we did not evaluate the relationship of malnutrition scores with inflammatory markers or with body mass index (BMI) because of the lack of height and weight in our database.

## Conclusion

The prevalence of malnutrition in diabetic patients with CAD was very high. Worsening malnutrition was associated with increased risk of all-cause mortality. Malnutrition assessment could allow clinicians to identify diabetic patients with CAD at elevated risk for mortality. Adequate assessment of nutritional status and necessary nutrition guidance can help improve the prognosis of diabetic patients with CAD.

## Data Availability

Not applicable.

## Abbreviations

ACS: Acute coronary syndrome
AMI: Acute myocardial infarction
AF: Atrial fibrillation
ACEI/ARB: Angiotensin-converting enzyme inhibitor/angiotensin receptor blocker
CONUT: Controlling Nutritional Status
CVD: Cardiovascular disease
CAD: Coronary artery disease
CHF: Congestive heart failure
CKD: Chronic kidney disease
CREA: Creatinine
CCB: Calcium channel blocker
DM: Diabetes mellitus
eGFR: Estimated glomerular filtration rate
FBG: Fasting blood glucose
HF: Heart failure
Hb: Hemoglobin
HbA1c: Glycosylated hemoglobin
HDL-C: High density lipoprotein cholesterol
hs-CRP: Hypersensitive C-reactive protein
LDL-C: Low density lipoprotein cholesterol
LVEF: Left ventricular ejection fraction
NRI: Nutritional Risk Index
OADs: Oral antidiabetic drugs
PCI: Percutaneous coronary intervention
PNI: Prognostic Nutritional Index
TC: Total cholesterol
TG: Triglycerides
T2DM: Type 2 diabetes mellitus
T1DM: Type 1 diabetes mellitus
2hPBG: 2 Hours postprandial blood glucose
